# Isoflurane Impairs Low-Frequency Feedback but Leaves High-Frequency Feedforward Connectivity Intact in the Fly Brain

**DOI:** 10.1523/ENEURO.0329-17.2018

**Published:** 2018-03-12

**Authors:** Dror Cohen, Bruno van Swinderen, Naotsugu Tsuchiya

**Affiliations:** 1 School of Psychological Sciences, Monash University, Melbourne 3800, Australia; 2 Queensland Brain Institute, The University of Queensland, Brisbane 4072, Australia; 3 Monash Institute of Cognitive and Clinical Neuroscience, Monash University, Melbourne 3800, Australia

**Keywords:** directed connectivity, Drosophila, feedback, general anesthesia, Granger causality, local field potentials

## Abstract

Hierarchically organized brains communicate through feedforward (FF) and feedback (FB) pathways. In mammals, FF and FB are mediated by higher and lower frequencies during wakefulness. FB is preferentially impaired by general anesthetics in multiple mammalian species. This suggests FB serves critical functions in waking brains. The brain of *Drosophila melanogaster* (fruit fly) is also hierarchically organized, but the presence of FB in these brains is not established. Here, we studied FB in the fly brain, by simultaneously recording local field potentials (LFPs) from low-order peripheral structures and higher-order central structures. We analyzed the data using Granger causality (GC), the first application of this analysis technique to recordings from the insect brain. Our analysis revealed that low frequencies (0.1–5 Hz) mediated FB from the center to the periphery, while higher frequencies (10–45 Hz) mediated FF in the opposite direction. Further, isoflurane anesthesia preferentially reduced FB. Our results imply that the spectral characteristics of FF and FB may be a signature of hierarchically organized brains that is conserved from insects to mammals. We speculate that general anesthetics may induce unresponsiveness across species by targeting the mechanisms that support FB.

## Significance Statement

In mammalian brains, neurons communicate through fast (high-frequency) feedforward (FF) and slow (low-frequency) feedback (FB) pathways. Various theories of consciousness propose that FB is crucial for the maintenance of consciousness. Here we used Granger causality (GC) analysis and multi-electrode recordings to investigate FB in the fly brain. Surprisingly, we found that the temporal characteristics of FF and FB are conserved in flies. Further, we found that isoflurane anesthesia preferentially reduced FB, consistent with results from mammalian studies. Our results imply that the mediation of FF and FB by higher and lower frequencies is a signature of hierarchically organized brains and that FB is a conserved, system-level target for general anesthetics.

## Introduction

Many complex networks including brains are hierarchically organized. In hierarchical systems information travels both from the bottom to the top of the hierarchy, known as feedforward (FF), and from the top to the bottom of the hierarchy, known as feedback (FB). It is only recently that the dynamic characteristics of neural FB and FF have been reported. For example, studies that investigated directed connectivity using Granger causality (GC) across the awake monkey visual hierarchy have shown that the alpha/beta and gamma bands preferentially mediate FB and FF, respectively (Bastos et al., 2014; van Kerkoerle et al., 2014). This pattern of low-frequency FB/high-frequency FF has also been reported across the visual (Michalareas et al., 2016) and auditory system (Fontolan et al., 2014) in awake humans. These studies imply that FB is mediated by lower frequencies than those that mediate FF.

The difference in frequency bands that mediate FB and FF is consistent with the functional explanation of these under the hierarchical predictive coding framework (Hohwy, 2013). According to predictive coding, compared to FF, FB necessarily operates at slower timescales because its main function is to provide prediction of incoming signals through the incorporation of memory and expectation. On the other hand, FF has to react to quickly changing sensory inputs, necessarily operating at faster timescales than FB. Note that these hypotheses may be true for all hierarchically organized biological brains, from insects to mammals, to the extent that these functional requirements are shared across species.

Anatomically, fly brains are hierarchically organized from peripheral sensory layers to central nuclei, which are responsible for more abstract computation (Paulk et al., 2013a; Shih et al., 2015; Haberkern and Jayaraman, 2016). Like mammals, wakeful insect brains also face functional challenges in adjusting fast sensory inputs based on prior experience on a slower time scale (Card and Dickinson, 2008; Combes, 2015; Kim et al., 2015; Mischiati et al., 2015). In addition, recent work suggests that the topological organization of the mammalian brain, which shares a number of similarities with the topological organization of the fly brain (Shih et al., 2015), influences the direction of information flow (Moon et al., 2015; Mejias et al., 2016; Moon et al., 2017). This evidence suggests that similar dynamic characteristics of FF and FB processing will be present in the insect brain.

A separate line of inquiry suggests that FB in particular is crucial for waking brain functions. A number of studies have demonstrated that general anesthetics preferentially reduce FB from frontal to posterior areas as measured using human scalp level electroencephalography (EEG; Lee et al., 2009; Ku et al., 2011; Boly et al., 2012; Jordan et al., 2013; Lee et al., 2013; Ranft et al., 2016). Although a subset of studies show differing results (Barrett et al., 2012; Nicolaou et al., 2012; Maksimow et al., 2014), the disrupted frontal-parietal connectivity has also been demonstrated using functional magnetic resonance imaging (fMRI; Hudetz and Mashour, 2016). In addition, general anesthetic suppression of FB has also been reported in rodents (Imas et al., 2006; Pal et al., 2016), ferrets (Wollstadt et al., 2017), and monkeys (Lamme et al., 1998; Papadopoulou et al., 2015). Note, however, that Wollstadt et al. (2017) show that FB (prefrontal cortex to V1) is reduced, but that this reduction may be a result of reduced “source information” within the prefrontal cortex. Also, Lamme et al. (1998) have shown that general anesthesia reduces contextual processing, which has been taken as an indirect support for the impairment of FB influences to V1.

Flies are also rendered unresponsive by general anesthetics and in similar concentrations to humans (van Swinderen and Kottler, 2014; Zalucki and van Swinderen, 2016). Indeed, the cellular and molecular machinery that makes up the nervous system is largely conserved across species (Littleton and Ganetzky, 2000), such that the cellular and molecular targets of general anesthetics are also likely to be conserved (Sonner, 2008; van Swinderen and Kottler, 2014; Zalucki and van Swinderen, 2016). Whether the conserved cellular and molecular targets of general anesthetics also translate to conserved system-level general anesthetic effects, such as reduced FB, is currently unknown.

Here, we investigated the dynamic characteristics of FB and FF in the small brains of fruit flies, *Drosophila melanogaster*. We analyzed directional connectivity in LFPs recorded from low-order peripheral structures and higher-order central structures. This showed that low frequencies (0.1–5 Hz) mediated FB from the center to the periphery, while higher frequencies (10–45 Hz) mediated FF in the opposite direction. Further, the general anesthetic isoflurane reduced FB while leaving FF intact. Our results imply that the preferential mediation of FB and FF by low and high frequencies, respectively, may be a signature of hierarchically organized brains and that general anesthetics induce unresponsiveness by targeting FB mechanisms.

## Materials and Methods

### Overview

We analyzed local field potentials (LFPs) data previously recorded from the brains of awake and anesthetized flies (Cohen et al., 2016). Whereas Cohen et al. (2016) focused on visually evoked responses, here we focus on the analysis of power, coherence, and GC of spontaneous LFPs, and how these are affected by isoflurane. In this section, we briefly recap the experimental setup. For the full experimental details, see Cohen et al. (2016).

**Figure 1.**
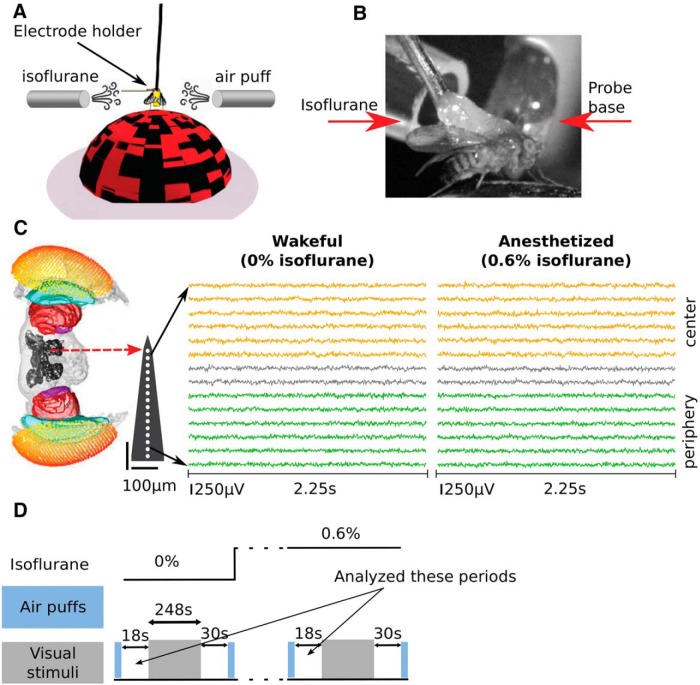
Experimental setup. ***A***, Flies were dorsally fixed to a tungsten rod and placed on an air-supported ball where they could freely walk. Isoflurane in different volumetric concentrations was delivered through a rubber hose. Air puffs were used to gauge flies’ responsiveness. A 16-contact electrode probe mounted on an electrode holder was inserted laterally from the left into the fly’s eye. Only the electrode holder is visible at the depicted scale. ***B***, A close-up view contralateral to the electrode insertion site showing the tethered fly, isoflurane delivery hose, and probe base. ***C***, Example of pre-processed LFPs data before anesthesia (0% isoflurane) from one fly. A standardized fly brain is shown for comparison (Paulk et al., 2013b, 2015). The electrode contacts are indicated by white dots (not to scale). For visualization the LFPs are shown subsampled to 250 Hz (analyzed at 1000 Hz). Six channels are grouped as peripheral, corresponding to the optic lobe (green), and another six channels are grouped as central, corresponding to the central brain (orange). ***D***, An experiment consisted of multiple blocks at different concentration of isoflurane (top black line). Each block proceeded with (1) a series of air puffs (light blue rectangles) followed by 18 s of rest; (2) presentation of visual stimuli (248 s); (3) 30 s of rest followed by a series of air puffs; and (4) isoflurane concentration change followed by 180 s of rest.

Thirteen female laboratory-reared *D. melanogaster* (Canton S wild type) flies (3–7 d after eclosion) were collected under cold anesthesia, tethered and positioned on an air-supported Styrofoam ball. Linear silicon probes with 16 electrodes separated by 25 μm (Neuronexus 3mm-25-177) were inserted laterally to the eye of the fly until the most peripheral electrode site was just outside the eye. This probe covers approximately half of the fly brain and records neural activity from both peripheral and central brain structures (Fig. 1*C*). A fine tungsten wire was inserted in the thorax and used as a reference electrode.

Isoflurane was delivered through a rubber hose connected to an evaporator (Fig. 1*A*,*B*). The effects of isoflurane were tested at concentrations of 0% and 0.6% (for details, see Cohen et al., 2016). An olfactory stimulus controller was used to deliver six air puffs to gauge the behavioral responsiveness of the flies in each concentration of isoflurane. Fly movement activity in response to the air puff was recorded with a camera. We used the video data to confirm that 0.6% isoflurane abolished the behavioral responsiveness and that behavioral responsiveness returned after recovery (Cohen et al., 2016).

An experiment consisted of several blocks, each at a different concentration of isoflurane. Each block started with the delivery of a series of air puffs, followed by ∼18 s of rest, which is the critical period of data for this paper. Visual stimuli were presented, lasting 248 s in total, followed by an additional 30 s of rest. A series of air puffs were delivered again and then the isoflurane concentration was changed. After 180 s of adjustment to the new isoflurane concentration the experimental block was repeated (Fig. 1*D*).

In Cohen et al. (2016), we focused on the analysis of the visually-evoked data, which corresponds to the 248 s period of visual stimuli presentation. Here we focused on an 18-s period before the presentation of visual stimuli (Fig. 1*D*).

This no-visual stimulation period is better suited for coherence and GC analysis than the visual stimulation period because the stimulus-evoked responses can result in erroneous interpretation of coherence and GC (Truccolo et al., 2002; Wang et al., 2008). We focused on the period before rather than after visual stimuli presentation because we reasoned the latter period may also reflect residual visual activity.

The datasets analyzed in this study are available from the corresponding authors on request.

### LFP pre-processing

LFPs were recorded at 25 kHz, downsampled to 1000 Hz, and the most peripheral electrode site was removed from the analysis. We rereferenced the remaining 15 unipolar channels by subtracting adjacent channels (bipolar rereferencing) to obtain a set of 14 differential signals which we refer to as “channels” throughout this paper (Bastos et al., 2014; Trongnetrpunya et al., 2016; Cohen and Tsuchiya, 2017).

We divided the 18-s period into eight consecutive epochs of 2.25 s. We removed line noise from each epoch and bipolar rereferenced channel using three tapers, a window of 0.75 s and a step size of 0.375 s using the rmlinesmovingwinc.m function from the Chronux toolbox (http://chronux.org/; Mitra and Bokil, 2007). We then linearly detrended and *z*-scored each channel and epoch by removing the mean and dividing by the standard deviation across temporal points within each epoch (Seth, 2010; Bastos et al., 2014). Figure 1*C* shows example of the resulting LFPs from one fly before (0% isoflurane) and during anesthesia (0.6% isoflurane).

### Analyzing power

For power analysis we linearly detrended the pre-processed LFPs without the *z*-scoring operation, because *z*-scoring obscures overall shifts in power when comparing pre- and postanesthesia. We calculated power for each pre-processed channel *i* (i = [1-14]), epoch *e* (1-8), *k%* isoflurane concentration (k = [0 (air), 0.6]) and frequency ω, $S_{ek}^{i}(\omega )$ over the 2.25-s epochs. Using three tapers, the half bandwidth was 0.89 Hz (Mitra and Pesaran, 1999). We further averaged power across eight epochs to obtain one estimate of power for each channel and isoflurane concentration $S_{k}^{i}(\omega )$.

We analyzed the average power across channels within the center $S_{k}^{C}(\omega )$ and periphery $S_{k}^{P}(\omega )$ in units of 10log10μV^2^, following the division of channels into peripheral channels=[1–6] and central channels=[9–14] as in Cohen et al. (2016)

$$\begin{array}{l} S_{k}^{P}(\omega )=\frac{1}{6}\overset{6}{\underset{i=1}{\sum }}S_{k}^{i}(\omega )\\ S_{k}^{C}(\omega )=\frac{1}{6}\overset{14}{\underset{i=9}{\sum }}S_{k}^{i}(\omega ) \end{array}$$

In the fly brain, the locations of the peripheral channels correspond to the primary visual systems while those of the central channels to higher-order structures (Paulk et al., 2013a; Shih et al., 2015; Haberkern and Jayaraman, 2016). In Cohen et al. (2016), we found that these two rough divisions explained different electrophysiological responses during the visual stimulation period.

We report the effect of isoflurane on power in the central brain as the difference in power between 0.6% and 0% in decibels (dB)

$$\Delta S_{0.6}^{C}(\omega )=S_{0.6}^{C}(\omega )-S_{0}^{C}(\omega )$$

Analogous derivations give the effect of 0.6% isoflurane on power in the periphery $\Delta S_{0.6}^{P}(\omega )$.

Previous work has shown that the fly heartbeat can manifest as low-frequency (2–4 Hz) oscillatory activity in central channels (Paulk et al., 2013b; Yap et al., 2017). Heartbeats are unlikely to affect the results presented here because we use bipolar rereferencing which eliminates common input to neighboring channels, as may be expected from a muscle artifact (Cohen and Tsuchiya, 2017). To further rule out heartbeat as a potential confound we also analyzed GC (see Non-parametric GC analysis) in our data after excluding three flies in which we detected potential heartbeat in central channels by visual inspection of the power spectra. The results of this analysis closely matched those obtained with all thirteen flies, indicating the any heartbeat contamination is unlikely to affect our results.

### Analyzing coherence

Coherence measures the strength of linear dependency between two signals in the frequency domain (Bendat and Piersol, 2000). It is defined as

$$C^{ij}(\omega )=\frac{abs{\left(CS^{ij}(\omega )\right)}^{2}}{S^{i}(\omega )S^{j}(\omega )}$$

$CS^{ij}(\omega )$ is the cross-spectrum between channels i and j, and $S^{i}(\omega )$ and $S^{j}(\omega )$ are the power spectra of channels i and j, respectively. To estimate coherence for each bipolar rereferenced channel pair *i* and *j* (i,j = [1-14]), epoch *e* (1-8) and *k%* isoflurane concentration (k = [0 (air), 0.6]) we used the multitaper method with nine tapers for each 2.25-s epoch, giving a half bandwidth of 2.22 Hz (Mitra and Pesaran, 1999). The auto and cross spectra for channels *i* and *j* at isoflurane concentration *k*% were averaged across the eight epochs to give one estimate of coherence at each isoflurane concentration.

We report center ($C^{C}$) and periphery ($C^{P}$) coherence as averaged across all non-neighboring center and periphery channel pairs

$$C_{k}^{C}(\omega )=\frac{1}{10}\overset{12}{\underset{i=9}{\sum }}\overset{14}{\underset{j=i+2}{\sum }}C_{k}^{ij}(\omega )$$

$$C_{k}^{P}(\omega )=\frac{1}{10}\overset{4}{\underset{i=1}{\sum }}\overset{6}{\underset{j=i+2}{\sum }}C_{k}^{ij}(\omega )$$

We exclude neighboring channels pairs because these show artefactual high coherence due to the bipolar rereferencing (for details, see Shirhatti et al., 2016; Cohen and Tsuchiya, 2017).

We report center-periphery coherence ($C^{CP}$) as averaged across all center-periphery pairs

$$C_{k}^{CP}(\omega )=\frac{1}{36}\sum_{i=1}^{6}\sum_{j=9}^{14}C_{k}^{ij}(\omega )$$

### Estimating the coherence bias

Because coherence is limited to the 0–1 range it has an inherent positive bias (Jarvis and Mitra, 2001). We used surrogate data statistics to estimate the empirical coherence bias in our data. The advantages of this method are (1) that it provides an empirical lower bound above which coherence may be considered significant and (2) that it accounts for potentially frequency-specific coherence biases sometimes observed for two time-series with similar power spectra (Faes et al., 2004).

For each of 13 flies we created surrogate data in which we randomized the phase of the signal while keeping the amplitude the same (Faes et al., 2004; Fasoula et al., 2013). We performed this 200 times for each channel (1–14), epoch (1–8) and isoflurane concentration ([0%, 0.6%]) resulting in surrogate data with the same power spectra as the original data but with near-zero cross spectra between channels. The surrogate data describes the null hypothesis that there is no coherence in the data.

To estimate the center, periphery and center-periphery coherence bias at *k*% isoflurane we calculated coherence as described above for each of the 200 surrogate data sets, and averaged the estimates across all 200 surrogates. We obtained the 95% confidence intervals by determining the 2.5 and 97.5 percentiles across the 200 surrogates for each fly.

When we compared the coherence bias in 0.6% and 0% isoflurane, we observed differences in some frequencies (0.1–5 Hz), but the magnitudes of the differences were very small (largest difference was 0.0038 at around 11 Hz for center coherence). Accordingly, our coherence results in 0.6% and 0% isoflurane with or without bias correction were nearly identical.

### Non-parametric GC analysis

In simple terms, a signal X is said to Granger-cause a signal Y if past values of X improve predictions of future values of Y. This notion of causality is based on modeling X and Y as autoregressive processes. GC can be estimated in either the time or frequency domain. The latter provides spectral decomposition of GC influences. We hypothesized that examining GC influences at different frequencies is critical because recent work that studied mammalian brains showed that different directional interactions were mediated by different frequencies (Bastos et al., 2014; van Kerkoerle et al., 2014; Michalareas et al., 2016).

GC can be estimated parametrically by fitting an autoregressive model to the data (Geweke, 1982, 1984; Chen et al., 2006). The drawback of this approach is that it requires selection of the model order. Alternatively, GC can be non-parametrically estimated directly from the spectral density matrix (Dhamala et al., 2008; Wen et al., 2013). The spectral density matrix for channels i and j at isoflurane concentration *k*% $Q_{k}^{ij}(\omega )$ is obtained by setting the diagonal elements to the auto-spectra and the cross-diagonal elements to the cross-spectra (Wen et al., 2013)

$$Q_{k}^{ij}(\omega )=\left(\begin{array}{cc} S_{k}^{i}(\omega ) & CS_{k}^{ij}(\omega )\\ CS_{k}^{ji}(\omega ) & S_{k}^{j}(\omega ) \end{array}\right)$$

We estimated the spectral density matrix using the same estimates of auto- and cross-spectra as those used for coherence analysis. To obtain Granger-causal influences from channel *i* to channel *j* at isoflurane concentration *k*% $f_{k}^{i\to j}(\omega )$ we first factorized the spectral density matrix using the *sfactorization_wilson*.m function and then estimated the Granger-causal influences using the *ft_connectivity_Granger.m* function. These functions are available from the FieldTrip toolbox (Oostenveld et al., 2011).

### Grouping FF and FB influences

In the fly brain, visual information enters through the retina and is sequentially processed by the lamina, medulla and lobula, collectively referred to as the optic lobe (Sanes and Zipursky, 2010; Paulk et al., 2013a). Visual projection neurons (VPNs) convey information from lower to higher-order visual processing centers, called optic glomeruli, which are located more centrally (Otsuna and Ito, 2006). Relatively little is known about computations in the optic glomeruli, but recent studies suggest a role in second-order motion processing (Zhang et al., 2013) and processing that mediates natural avoidance behaviors (Wu et al., 2016). Specific optic glomeruli, such as the ventrolateral protocereberum (Ito et al., 2014), send projections back to the lobula and medulla, as well as reciprocated projections to other central structures (Otsuna and Ito, 2006; Shih et al., 2015). Our simple summary of this connectome is that visual information enters in the periphery and sequentially conveyed through FF connections toward more central optic glomeruli, which convey FB back to the optic lobe.

We emphasize that our definition of FF and FB is based only on this anatomic organization. Because we did not manipulate bottom-up or top-down processing through stimulus characteristics or task demands, we do not claim that our FF and FB estimates directly reflect bottom-up sensory processing or top-down cognitive functions.

To estimate overall FF GC influences at k% isoflurane $f_{k}^{FF}(\omega )$ we averaged across all GC influences $f_{k}^{i\to j}(\omega )$ from the periphery to the center

$$f_{k}^{FF}(\omega )=\frac{1}{36}\overset{6}{\underset{i=1}{\sum }}\overset{14}{\underset{j=9}{\sum }}f_{k}^{j\to i}(\omega )$$

where channel 1 is the most peripheral and channel 14 is the most central. Analogous derivations give FB GC influences

$$f_{k}^{FB}(\omega )=\frac{1}{36}\sum_{i=1}^{6}\sum_{j=9}^{14}f_{k}^{j\to i}(\omega )$$

### Estimating the Granger causal influences bias

GC influences are always positive and therefore have a positive bias. We checked whether the GC bias differed between FF and FB or across isoflurane concentrations. To estimate the GC bias, we used the same procedure for the estimation of the coherence bias through phase randomization. This procedure results in 200 estimates of FF and FB GC influences for each fly, at each isoflurane concentration *k*%. We obtained the final estimate of the FF and FB GC bias by averaging across all 200 surrogates.

The differences between FF and FB GC biases at each isoflurane concentration, as well as the differences in FF and FB GC biases between isoflurane concentrations were negligible. The maximum difference in group average bias was 0.0008 (difference between FF and FB at 0.6% isoflurane at around 5 Hz). The difference in GC bias averaged across 0.1–45 Hz were all negligible: 0.00006 (between FF and FB in 0% isoflurane), 0.0002 (between FF and FB in 0.6% isoflurane), 0.0002 (between 0% and 0.6% isoflurane for FF), and 0.0001 (between 0% and 0.6% isoflurane for FB).

### Directed asymmetry index (DAI) analysis

Because the magnitude and variance of GC influences are dependent on the frequencies, some form of normalization is necessary for statistically comparing them. For this purpose, we adopt the DAI (Lee et al., 2013; Bastos et al., 2015; Michalareas et al., 2016), which is defined as

$$DAI=\frac{FF-FB}{FF+FB}$$

The numerator of the DAI captures the predominant direction of the GC influence. The denominator provides normalization for the total GC influences at a given frequency. DAI allows comparisons across conditions and frequencies where there may be an overall net increase or decrease in GC influences.

Given our enumeration of channels (i.e., *i->j* indicates FF influences if *i < j*), the DAI between channel *i* and *j* at isoflurane concentration *k*% is given by

$$DAI_{k}^{ij}(\omega )=\frac{f_{k}^{i\to j}(\omega )-f_{k}^{j\to i}(\omega )}{f_{k}^{i\to j}(\omega )+f_{k}^{j\to i}(\omega )},i\in [1-6],j\in [9-14],$$

We report the DAI averaged over all center-periphery channel pairs

$$DAI_{k}(\omega )=\frac{1}{36}\sum_{i=1}^{6}\sum_{j=9}^{14}DAI_{k}^{ij}(\omega )$$

### Statistical analysis

To test for statistical differences between 0% and 0.6% isoflurane across frequencies (power, coherence, GC, and DAI spectrums) we used randomization-based, non-parametric statistical tests with cluster-based multiple comparison correction (Maris and Oostenveld, 2007). Each of the 13 flies provided one spectrum for each condition (0% and 0.6% isoflurane). We then performed paired t tests across 13 flies at each frequency to obtain t scores. If we found a cluster of continuous frequencies at *p* < 0.05 level, then we treated them as a first-level significant cluster. As a second-level statistic, we used the sum of the t scores across frequencies within each cluster. We then preformed 8191 randomizations corresponding to all possible randomizations of thirteen flies across the two conditions (2^–13^ – 1 = 8191) and calculated the second-level statistic for each randomization. From each randomization, the largest second-level statistics was chosen and used for constructing a randomized distribution. We compared the observed second-level statistics against the randomized distribution to obtain *p* values.

**Figure 2.**
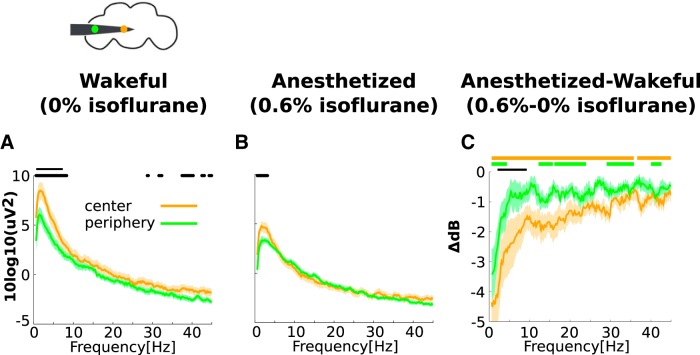
Isoflurane reduces power in the center and periphery. ***A***, Group average power in the center (orange) and periphery (green) in 0% isoflurane (before any anesthesia). A schematic representation of the fly head and inserted electrode showing the estimated positions of one central (orange) and one peripheral (green) channel is depicted above. Black dots and solid line mark uncorrected and corrected significant differences between center and periphery power at the *p* < 0.05 level, respectively (see Materials and Methods). ***B***, Same as ***A*** in 0.6% isoflurane. ***C***, The effect of isoflurane on power in the center and periphery, obtained by subtracting values in 0% isoflurane from values in 0.6% isoflurane. Negative values indicate that isoflurane reduced power for both the center and periphery. For central channels, orange thick lines above indicate significant reductions due to isoflurane (corrected, *p* < 0.05). For peripheral channels, green thick lines above indicate corresponding significant reductions (corrected, *p* < 0.05). Greater reductions in power in the center compared to the periphery are indicated by the black line (corrected, *p* < 0.05). Shaded area represents SEM across flies (*N* = 13) for all panels.

**Figure 3.**
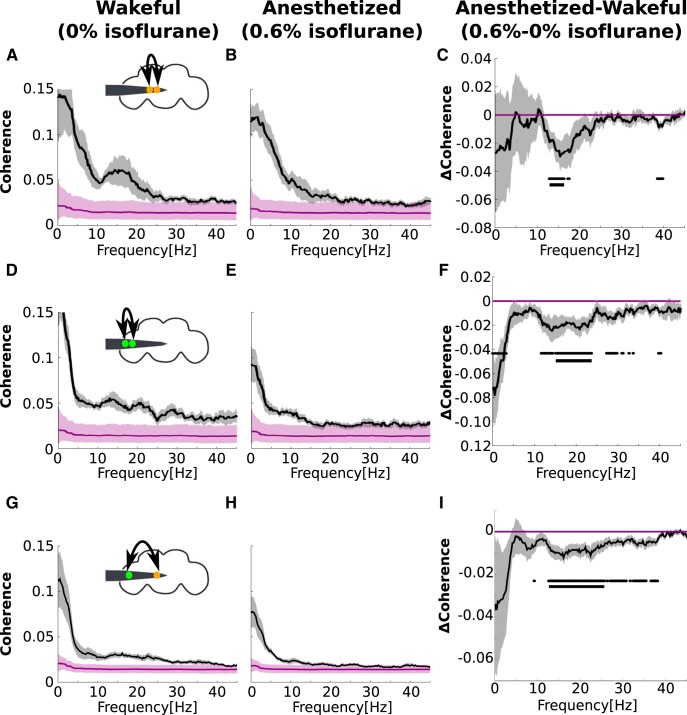
The effect of isoflurane on center, periphery, and center-periphery coherence. ***A***, Coherence between electrodes in the central brain in 0% isoflurane (black line). Gray shades represent SEM across flies (*N* = 13). Purple line and shades represents the bias level and its 95% confidence intervals. A schematic representation of the fly head and inserted electrode showing the estimated positions of two central channels is depicted above. ***B***, Same as **a** in 0.6% isoflurane. ***C***, The effect of isoflurane on center coherence, obtained by subtracting values in 0% isoflurane from values in 0.6% isoflurane. Dots and solid lines below the effect of isoflurane (***C***, ***F***, ***I***) indicate significant reductions (*p* < 0.05) uncorrected and corrected, respectively, for multiple comparisons. The same format is repeated in ***D–F*** for within-periphery coherence and ***G****–****I*** for center-periphery coherence.

**Figure 4.**
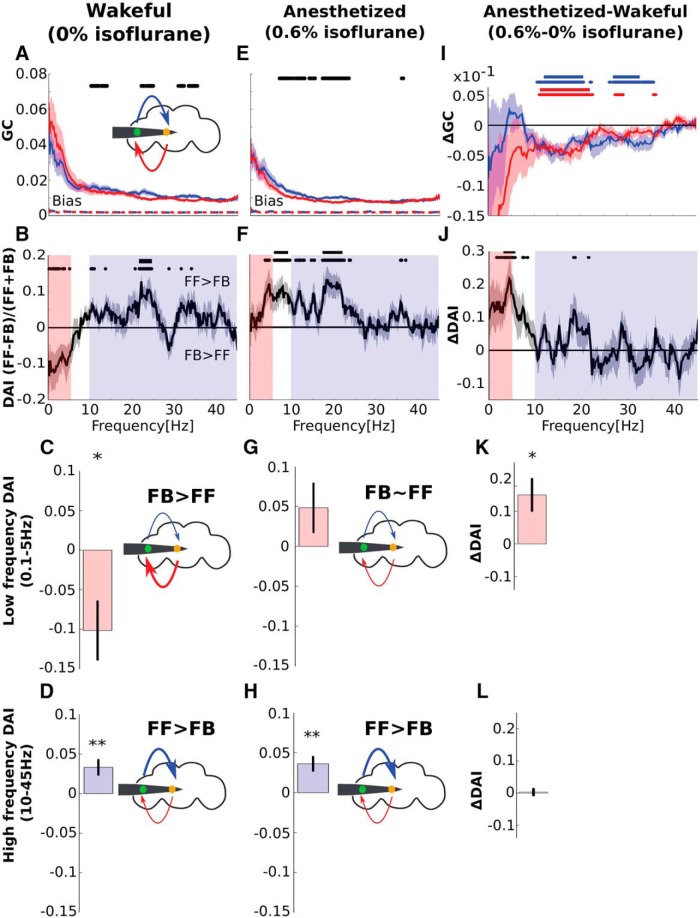
Isoflurane reduces low-frequency (0.1–5 Hz) FB in the fly brain. ***A***, Group level FF (blue) and FB (red) GC influences before any anesthesia (0% isoflurane). Black dots mark significant differences between FF and FB at the *p* < 0.05 level (uncorrected). The FF and FB GC bias levels (see Materials and Methods) are shown as dashed red and blue lines, which almost completely overlap near zero. ***B***, Group level DAI before any anesthesia (0% isoflurane). Positive values indicate FF > FB and negative values indicate FF < FB. Black dots and solid line mark significant uncorrected and corrected differences from zero at the *p* < 0.05 level. Low (0.1–5 Hz) and high (10–45 Hz) frequencies are indicated by the pale pink and purple shadings. ***C***, ***D***, Group level DAI averaged for low (0.1–5 Hz; ***C***) and high (10–45 Hz; ***D***) frequencies before any anesthesia (0% isoflurane). Negative and positive DAI values indicate that FB > FF for low frequencies and that FB < FF for high frequencies. The fly head schemas above the graphs depict this as thicker FF (red, ***C***) and FB (blue, ***D***) arrows. ***E–H***, Same as ***A–D*** for 0.6% isoflurane. ***I***, The effect isoflurane on FF (blue) and FB (red) GC, obtained by subtracting values in 0% isoflurane from 0.6% isoflurane. Blue (and red) dots and solid line mark significant uncorrected and corrected reductions in FF (and FB) GC (*p* < 0.05). ***J***, The effect of isoflurane on DAI, obtained by subtracting values in 0% isoflurane from 0.6% isoflurane. Black dots and solid line mark significant uncorrected and corrected differences from zero (*p* < 0.05). ***K***, ***L***, The effect of 0.6% isoflurane on low (***K***) and high frequencies (***L***), obtained by subtracting values in 0% isoflurane (***C***, ***D***) from 0.6% isoflurane (***G***, ***H***). * and ** indicate significant differences from zero at the *p* < 0.05 and *p* < 0.01 level, respectively (group-level permutation tests, see Materials and Methods). Error bars and shaded areas represent SEM across flies (*N* = 13) for all panels.

Figures 2–4 show the results from the first-level (uncorrected) t tests as well as the second-level cluster-based statistics, which corrects for multiple comparisons across frequencies. When we compared DAI spectrums against zero (Fig. 4*B*,*F*,*J*), we randomized the FF and FB labels for each fly (Michalareas et al., 2016). For testing whether the DAIs averaged across low and high frequencies were different from zero (Fig. 4*C*,*D*,*G*,*H*) we randomized the FF and FB labels and used the group mean as the test metric. For testing the effect of isoflurane on the DAIs averaged across low and high frequencies (Fig. 4*K*,*L*), we randomized the isoflurane concentrations labels and used the group mean difference between the conditions as the test metric.

## Results

### Isoflurane reduces spontaneous power in the center and periphery

We investigated the dynamic characteristics of FB and FF and how these are affected by anesthesia by analyzing LFPs recorded from the center and periphery of awake and anesthetized fly brains (Fig. 1). Before analyzing FB and FF we first assessed whether neural activity is distinguishable across the center and periphery. To do this we analyzed the power of central and peripheral channels separately (Fig. 2*A*). We found that power in both the center and periphery was highest at low frequencies and decreased as frequency increased. In low frequencies, power in the center was significantly greater than power in the periphery, demonstrating that neural activity was indeed distinguishable across these two anatomic areas (Fig. 2*A*). At a concentration of 0.6% isoflurane, the flies were rendered unresponsive, as probed by air puffs (Cohen et al., 2016). We found that during anesthesia, power in both the center and periphery was reduced (0.6% isoflurane; Fig. 2*B*). For the center, power was significantly reduced across almost all frequencies tested. For the periphery the reduction was smaller and only significant in a subset of the frequencies tested (Fig. 2*C*). The reduction in power in the center was greater than the periphery for low frequencies. These observations are consistent with an overall suppression of neural activity but also suggest that this suppression is more consistent in the higher-order central structures of the fly brain.

### Isoflurane reduces center, periphery, and center-periphery coherence

Next, we assessed the effects of isoflurane on local and global processing. To do this, we analyzed coherence, a spectral measure of linear dependency between signals (Bendat and Piersol, 2000). To assess the effects on local processing, we analyzed coherence within the center and periphery. We calculated center and periphery coherence using all non-neighboring center and periphery channel pairs (for details, see Materials and Methods).

Within-center coherence was highest for low frequencies and showed an additional peak around 10–20 Hz (Fig. 3*A*). Because coherence is confined to 0–1, it is inherently biased. Using surrogate data methods (see Materials and Methods), we found that the coherence was above the bias level for 0.1–45 Hz (Fig. 3*A*). We repeated the coherence analysis during anesthesia and found that the coherence remained above the bias for all frequencies (Fig. 3*B*). Isoflurane significantly reduced within-center coherence around 15 Hz (Fig. 3*C*).

Similarly, within-periphery coherence was highest for low frequencies, decreased as frequency increased and remained above the bias for 0.1–45 Hz (Fig. 3*D*). Unlike within-center coherence, however, within-periphery coherence had no prominent peak in the 10–20 Hz range (Fig. 3*D*). During anesthesia all frequencies remained above the bias (Fig. 3*E*). Isoflurane significantly reduced within-periphery coherence in the 15–25 Hz range (Fig. 3*F*).

Finally, we assessed the effects of anesthesia on more global processing by calculating center-periphery coherence, which we calculated using all center-periphery channel pairs (for details, see Materials and Methods). We found that similar to center and periphery coherence, center-periphery coherence was highest for low frequencies, decreased with increasing frequency and remained above the bias for 0.1–45 Hz (Fig. 3*G*). During anesthesia, low-frequency coherence remained above the bias, whereas high-frequency coherence was not distinguishable from the bias (Fig. 3*H*). Isoflurane significantly reduced center-periphery coherence around the 10–25 Hz range.

In sum, our coherence results indicate that isoflurane impaired localized processing within the center and periphery as well as global processing between the center and periphery.

### Low- and high-frequencies preferentially mediate FB and FF, respectively

Finally, we analyzed directional connectivity. To dissect the directionality we used GC analysis (Dhamala et al., 2008; Wen et al., 2013; Seth et al., 2015). We analyzed Granger causal influences from the periphery to the center as FF GC influences, and influences from the center to the periphery as FB GC influences (Fig. 4). We found that both FF and FB GC were well above any bias level in the GC measure. FB GC appeared greater than FF in lower frequencies (Fig. 4*A*). However, as is apparent in Figure 4*A*, the variance of spectral GC influences also appears larger in lower frequencies. Thus, before comparing FF and FB GC we corrected for these statistical characteristics. To do this, we employed a normalized measure known as the DAI (Lee et al., 2013; Bastos et al., 2014; Michalareas et al., 2016). The DAI is defined as

$$DAI=\frac{FF-FB}{FF+FB},$$

which is positive when FF is stronger than FB and vice versa. For the DAI the variance was uniform across all frequencies (Fig. 4*B*).

During wakefulness (0% isoflurane), the DAI was negative for lower frequencies (∼0.1–5 Hz), indicating that FB is stronger than FF. For higher frequencies (10–45 Hz) the DAI is predominantly positive, indicating that FF is stronger than FB. To summarize these results, we averaged the DAI for low (0.1–5 Hz; Fig. 4*C*) and high (10–45 Hz; Fig. 4*D*) frequencies separately. We found that in awake flies FB is stronger than FF for low frequencies (0.1–5 Hz; Fig. 4*C*, *p* < 0.05, group-level permutation tests, see Materials and Methods) and that FB is weaker than FF for high frequencies (10–45 Hz; Fig. 4*D*, *p* < 0.01). This global pattern of results, that is, the dominance of FB in lower frequencies and the dominance of FF in higher frequencies, is generally consistent with what has been reported in the awake mammalian brain (Bastos et al., 2014; Fontolan et al., 2014; van Kerkoerle et al., 2014; Michalareas et al., 2016).

### Isoflurane reduces FB

Next, we examined the effect of isoflurane on FB and FF GC. In humans, general anesthetics have been shown to preferentially reduce FB (Lee et al., 2009; Boly et al., 2012; Jordan et al., 2013; Lee et al., 2013; Ranft et al., 2016). In our data, we found that during anesthesia both FF and FB influences were reduced in magnitude (Fig. 4*E*). The DAI, which normalizes the magnitude and variance across frequencies, was no longer negative for low frequencies but remained positive for high frequencies (Fig. 4*F*). This meant that during anesthesia the dominance of FB over FF was lost in low frequencies (Fig. 4*G*), and that the dominance of FF over FB in high frequencies remained intact (Fig. 4*H*). Statistical testing confirmed a frequency- and direction-specific effects on GC (Fig. 4*I*), resulting in an increase in the DAI in low frequencies (Fig. 4*J*,*K*; 0.1–5 Hz, *p* < 0.05). There was no change in the DAI in high frequencies (Fig. 4*L*; 10–45 Hz, *p* = 0.8).

## Discussion

In hierarchically organized brains, dedicated mechanisms may be necessary for routing information in an effective manner. In particular, the hallmark dynamics of hierarchical brains may be the modulation of lower-level, fast responses to the changing environment by higher-level, slower processing that incorporate prior information and expectation (Tani and Nolfi, 1999; Friston, 2010; Bastos et al., 2012; Hohwy, 2013). We found that, like human and monkey brains, FB and FF influences are mediated by lower and higher frequencies in fly brains. This result is surprising given the obvious differences in the number of neurons [∼10^5^ neurons in flies (Chiang et al., 2011) and 10^11^ neurons in humans (Herculano-Houzel, 2009)] and gross neuroanatomy between these brains. However, a recent study that analyzed the fly connectome has demonstrated a number of organizational similarities between the fly and mammalian brains, such as hierarchical structure and other graph theoretical characteristics (e.g., small-world and rich-club organization; Shih et al., 2015). These structural similarities may translate to similar dynamic characteristics of FB and FF, as recently demonstrated by analytical and computational studies (Moon et al., 2015, 2017; Mejias et al., 2016).

From a functional viewpoint, the dynamical characteristics of FF and FB may also serve similar roles across species, and possibly even hierarchically organized robots (Yamashita and Tani, 2008). Predictive coding suggests that “predictions” travel in the FB direction while “prediction errors” travel in the FF direction (Tani and Nolfi, 1999; Friston, 2010; Bastos et al., 2012; Hohwy, 2013). Because the updating of predictions depends on gathering prediction errors over time, predictions are inherently mediated through slower time scales (lower frequencies) than prediction errors (Bastos et al., 2012; Bastos et al., 2015; Michalareas et al., 2016). In insects, a number of recent studies have demonstrated predictive behaviors and begun to investigate their neural correlates (Card and Dickinson, 2008; Combes, 2015; Kim et al., 2015; Mischiati et al., 2015). Our finding that FB and FF influences are mediated by low and high frequencies in the fly brain is consistent with the idea that these animals are also engaging in a type of predictive coding. Predictive coding can be formulated under the more fundamental framework of free-energy minimization (Friston, 2010), which may apply to biological systems more broadly (Friston, 2012).

We found that isoflurane reduced both local coherence within the center and periphery as well as global coherence between the center and periphery. For directional connectivity, we found that both FF and FB were reduced, but that for low frequencies isoflurane preferentially reduced FB. A number of human studies have demonstrated that general anesthetics preferentially reduce frontal to posterior FB, as measured using EEG (Lee et al., 2009, 2013; Boly et al., 2012; Jordan et al., 2013; Ranft et al., 2016). Interestingly, a recent study using magnetoencephalography (MEG) found that such anterior to posterior (FB) influences are mediated by lower frequencies than those that mediated posterior to anterior (FF) influences (Hillebrand et al., 2016).

Our findings in flies parallel results previously reported for mammals. However, we acknowledge important limitations in regards to this comparison. In particular, we presented analyses of spontaneous, not sensory-evoked, LFPs. In the absence of a sensory signal to transmit, we do not know if the FF and FB we detected signal bottom-up and top-down processing. In this sense, cognitive processing or information transfer was not directly assessed. In Bastos et al. (2014), the presentation of a stimulus was necessary for clearly recruiting FF influences, which were interpreted as signaling bottom-up processing. While we did record data from periods of visual simulation (for details, see Cohen et al., 2016), these data were dominated by the stimulus frequency, violating the assumption of GC. It remains possible that in an experiment that uses sensory stimulation to engage bottom-up processes (yet left the data suitable for GC analysis), we would have detected more pronounced FF signaling and that this signaling would in fact be reduced by general anesthetics.

We also emphasize that not all general anesthetic effects found in mammals translate to flies. Indeed, one of the clearest system-wide effects of general anesthetics (as well as sleep) is an increase in low-frequency (0.1–4 Hz) power (Steriade et al., 1993; Amzica and Steriade, 1998; Chauvette et al., 2011; Murphy et al., 2011; Lewis et al., 2012; Purdon et al., 2013, 2015). Such an increase has not been reported for insects (van Swinderen, 2006; Kirszenblat and van Swinderen, 2015), and we did not observe this here (the results did not change with longer time window (18 s) and/or using a unipolar reference scheme [data not shown]). Interestingly, a recent study reported that sleep *induction* in flies is accompanied by an increase in 7–10 Hz LFPs power (Yap et al., 2017). However, this was not observed for pharmacologically induced sleep, so any relevance to general anesthesia remains unknown. Further studies of population-level dynamics in flies will be required to fully understand the similarities between the way general anesthetics affect the fly and mammalian brain. Our study is a step in this direction, and suggests that low-frequency FB may be a system-wide target process for general anesthetics that is conserved across species.

## Synthesis

Reviewing Editor: Marlene Bartos, University of Freiburg Decisions are customarily a result of the Reviewing Editor and the peer reviewers coming together and discussing their recommendations until a consensus is reached. When revisions are invited, a fact-based synthesis statement explaining their decision and outlining what is needed to prepare a revision will be listed below. The following reviewer(s) agreed to reveal their identity: Andre Bastos, George Mashour

Reviewer 1:

In this study, Cohen et al report effects of Isofluorane on fly brain dynamics. The changes in top-down beta are worthwhile to report and interesting. However, I am concerned about the lack of slow oscillations - normally a highly conserved signal of anesthesia across many mammalian species. I am also concerned that the lack of bottom-up gamma may be a result of not doing the analysis in a time window that contains sensory stimulation.

In figure 1, experimental setup, the raw traces show no obvious oscillation during anesthesia. In fact, the authors report that power at all frequencies decreases under isofluorane anesthesia. This is strange, as most papers reporting effects of anesthesia in mammals (rodents, monkeys, humans), report the emerge of slow waves (0.1-1 Hz) and delta (2-4 Hz) during loss of consciousness.

This raises the question of whether fundamentally different systems-level mechanisms are responsible for anesthesia effects in flies, and whether there is sufficient construct validity between flies and mammals to generalize these effects.

I suggest the following analyses to investigate the possibility that certain parts of the analysis pipeline might have removed the slow activity from their data:

1. Please re-run the power analysis on the unipolar data. If the slow frequency activity is highly synchronous across the brain, the bipolar referencing might have removed it.

2. Increase the time window of analysis from ~2 seconds to ~10-20 seconds. If there is a slow oscillation around 0.1 Hz-2 (as we would expect from mammalian studies of anesthesia) than a 10-20second window would contain at least a few cycles of the oscillation. Note that for beta and gamma, the window size the authors chose is appropriate.

If the analyses does not reveal the presence of a slow wave during anesthesia, this does question the validity of these results, and the utility of the fly model for anesthesia. In this case I would recommend changing the title to “Isoflurane impairs low frequency feedback but leaves high frequency feedforward information intact in the fly brain” and also emphasizing in the discussion (more than they have already done) that the general patterns of anesthesia that are being studied here are highly different from the system wide effects seen in mammals, and that therefore the effects seen may not generalize to the mammalian brain.

Granger analysis:

The changes in top-down activity with beta during isofluorane are very interesting and worthwhile to report. In addition, the authors make the additional claim that bottom-up interactions are unchanged. However, since the analysis was performed during periods of no sensory stimulation, there is no real sense in referring to ‘bottom-up’ activity. In other words, bottom-up influences are expected to only matter when there is some kind of sensory signal to transmit. Previous studies of hierarchical influences have reported bottom-up gamma in the presence of visual/auditory stimulation (Bastos et al., van Kerkoerle et al., Fontolan). Indeed, in one of these studies (Bastos et al.), there was only evidence of bottom-up gamma in the presence of visual stimulation (in its absence, feedforward/feedback Granger causality in the gamma range was indistinguishable). Therefore, I would like to see whether the air puffs or sensory stimuli evoke an induced oscillatory response that is signaled in the feedforward direction. I understand the authors' concerns regarding the application of GC to event-related data, as the change in the mean/variance (ERP) violates the stationarity assumption of Granger. To mitigate this, the authors could try the following:

1) Subtract the ERP that is generated from the sensory stimulus from the data to get the ‘induced’ power, and perform GC on this.

2) Perform GC on the time window immediately after the stimulus, when the bulk of the event-related potential (ERP) has already decayed back to baseline levels (usually about 300-500 ms after stimulus onset).

3) Perform GC on the time window that contains the stimulus, and account for the non-stationary effects of the ERP using a moving time window approach, as outlined by Mingzhou Ding, Steven L Bressler, Weiming Yang, Hualou Liang (in: “Short-window spectral analysis of cortical event-related potentials by adaptive multivariate autoregressive modeling: data preprocessing, model validation, and variability assessment”).

4) Yet another possibility, which would involved additional experiments (but is also likely the most principled), is to use a form of visual stimulation which is unchanging (or minimally changing) in time - such as a grating, or a change in overall luminance that would be expected to be encoded by fly neurons. In the presence of a continuous visual stimulus, gamma oscillations are normally seen (in mammals) and although their power and frequency does change in time, the overall variance is stable - or at least more stable, rendering the assumption of non-stationarity more credible for a GC analysis.

Reviewer 2:

Cohen and colleagues report the remarkable observation that the brain of Drosophila exhibits (1) hierarchical organization with feedback and feedforward connectivity patterns that functionally parallel the mammalian brain, and (2) the feedback connectivity is selectively susceptible to the effects of a clinically-relevant anesthetic, suggesting--again, like in studies of humans and rodents--a role in conscious processing.

Comments:

1. Title- you might specify ‘Drosophila’ and ‘brain’ in the title. In order to be more conservative, you could also replace ‘information’ with ‘connectivity.’

2. Abstract- when you note feedback suppression by anesthetics, you should specify that this occurs in multiple mammalian species.

3. Introduction, 4th paragraph- when you discuss feedback suppression, you can also note that it occurs in multiple species, such as rodents (Imas et al, Neurosci Lett. 2005 Oct 28;387(3):145-50.; Pal et al, Anesthesiology. 2016 Nov;125(5):929-942.), ferrets (Wollstadt et al, PLoS Comput Biol. 2017 Jun 1;13(6):e1005511), and monkeys (Papadopoulou et al, Brain Topogr. 2015 PMID: 26350398). You could also cite Victor Lamme's earlier work on LOCAL recurrent processing in visual cortex, with feedback also preferentially suppressed by anesthetics.

4. Introduction, same paragraph: It is true that there have been analyses with differing results. However, the increases in linear-based connectivity measures are not consistent with multiple fMRI studies demonstrating disrupted frontal-parietal connectivity.

5. Methods- under the LFP section, I think you meant ‘rereferencing’ instead of ‘rerereferencing’

6. Results, coherence section- did you analyze peripheral-peripheral or central-central connectivity to assess changes in local synchrony?

7. Discussion- you might discuss Moon et al, PLoS Comput Biol. 2015;11(4):e1004225 and Moon et al, Sci Rep. 2017;7:46606. These studies help frame how the underlying network topology (structural or functional) can define directed connectivity patterns across different species or different states.

8. You should have a Limitations section, which includes the fact that cognitive processing or information transfer was not directly assessed.

9. General point on the manuscript is that the lower-order nature of the peripheral nodes and higher-order nature of the central nodes is critical to the scientific premise. You might bolster the introduction and discussion to really support that.
